# Demografia, saúde e emigração em Angola (c.1890-1945): uma perspetiva comparativa e transimperial

**DOI:** 10.1590/S0104-59702023000100020

**Published:** 2023-05-15

**Authors:** Cláudia Castelo

**Affiliations:** i Professora auxiliar, Departamento de História e Filosofia das Ciências/Faculdade de Ciências/Universidade de Lisboa. Lisboa – Portugal claucastelo@hotmail.com

Samuël Coghe, pesquisador pós-doutoral em história global na Universidade de Berlim, tem estudado o colonialismo europeu em África, em particular o controlo de doenças, a saúde pública, as políticas populacionais e, mais recentemente, o gado como mercadoria imperial. Os resultados das suas pesquisas têm sido publicados em revistas científicas com elevado fator de impacto, como as *Social History of Medicine, Journal of Contemporary History* ou *Medical History* .

Esse livro, baseado na tese de doutoramento do autor, defendida em 2014 no European University Institute, em Florença, corresponde a um exigente processo de maturação e revisão científica. O resultado é um estudo inovador que contribui para o avanço do conhecimento sobre Angola na primeira metade do século XX, nas vertentes da saúde, da demografia e da emigração, e o colonialismo português coevo. Porém, é muito mais do que isso. Inscrito numa história global que extravasa os quadros nacionais irá, seguramente, afirmar-se como referência na historiografia sobre políticas populacionais na África colonial.

Escrita com elegância e clareza, e editada com esmero na coleção “Global Health Histories”, da prestigiada Cambridge University Press, a obra inclui um índice remissivo e um conjunto de mapas, tabelas, reproduções de documentos e imagens que acrescentam informação ao texto e, no caso das fotografias, a presença da experiência humana. Nota-se uma verdadeira articulação entre os seis capítulos, além do encadeamento cronológico, o que ajuda a progressão na leitura e o entendimento dos argumentos do autor. Esses são sintetizados em conclusões parcelares no fim de cada capítulo e numa conclusão geral. Um epílogo remete-nos ao período temporal seguinte, entre o fim da Segunda Guerra Mundial e a independência de Angola (1945-75), marcado pelo *boom* da fixação de colonos brancos e pela persistência da ideia de um vasto e rico território fracamente povoado, quando noutras paragens se adensavam as preocupações com o sobrepovoamento.

À literatura sobre a história da doença do sono nas colónias portuguesas em África ( Amaral, 2012 ; Havik, 2014 ; Varanda, 2014 ), Coghe (2022) junta o olhar sobre as consequências do surto epidémico da década de 1890 em Angola: por um lado, a enorme ansiedade com o despovoamento do território e, por outro, as mudanças nas políticas populacionais do Estado colonial português que aquelas ansiedades espoletaram na transição para o século XX. Vista como a causa de uma significativa perda populacional e um perigo para a economia, devido à falta de mão de obra e à correlacionada diminuição da produção agrícola e do comércio, a doença era também encarada como uma ameaça à legitimidade do colonialismo português. Nesse contexto, a intervenção médica tornou-se uma necessidade demográfica e económica, um imperativo nacional e internacional e uma oportunidade para a realização de pesquisas científicas *in loco* , que tiveram lugar na primeira década do século XX.

Deborah Neill (2012) havia revelado a importância das redes de colaboração entre especialistas dos impérios coloniais alemão, belga, britânico e francês na tentativa de conhecer e controlar a doença do sono e como a pesquisa a seu respeito contribuiu para a institucionalização da medicina tropical na Europa. Esse livro avança uma história imbricada da afirmação da medicina tropical e da cooperação interimperial em África a partir do império português e da sua colónia de Angola, caso que tem passado despercebido na historiografia internacional. Coghe mostra que, dadas as dificuldades de implantação do novo conhecimento biomédico no terreno, não se pode falar numa estratégia de controlo única em Angola, mas de respostas fragmentárias. Além disso, ao invés de Daniel R. Headrick (2014) , defende a inexistência de um “estilo nacional” de combate à doença do sono, dada a variação nas formas de lidar com a doença no seio do império português, em função de condições locais.

O programa de saúde dirigido aos africanos, iniciado em 1926, no contexto da ditadura militar e depois do Estado Novo português, denominado Assistência Médica aos Indígenas (AMI), é estudado em profundidade pela primeira vez, com a preocupação de iluminar os debates que precederam seus estabelecimento, estrutura e objetivos, e ainda os constrangimentos estruturais que acabaram por limitar a sua expansão na década de 1930. São colocados em evidência o papel do médico e alto funcionário colonial António Damas Mora e dois momentos de aprendizagem interimperial que reverteram para a criação da AMI: a primeira conferência da África ocidental sobre medicina tropical (Luanda, 1923), organizada por Damas Mora; e a viagem de estudo que Mora e João Augusto Ornelas fizeram à África ocidental em 1926. Uma das mais-valias do livro é demonstrar como a história da AMI e das medidas contra a doença do sono foram profundamente determinadas por processos de comparação e trocas interimperiais, o que permite desmontar a narrativa do excepcionalismo do colonialismo português.

Ao descortinar o papel dos médicos como “demógrafos de campo”, assunto em grande medida inédito, Coghe expõe a agenda transformadora daqueles actores: usaram os dados que recolhiam (em circunstâncias difíceis e com precisão duvidosa), que apontavam para uma estabilidade da população, para legitimar uma reorientação da AMI no sentido da saúde infantil. Percebemos como essa mudança se operacionalizou, olhando para as intervenções destinadas a combater a mortalidade materno-infantil nos anos 1920 a 1940, em conexão com os debates e as políticas seguidas na metrópole e noutras colónias africanas: estabelecimento de maternidades estatais e a formação de parteiras angolanas; dispensários em meios urbanos, fruto da filantropia; e as maternidades e o treino de parteiras africanas por iniciativa das missões protestantes no universo rural. Embora esses esquemas correspondessem a uma visão negativa das mães africanas, nas quais se pretendia incutir a “arte da maternidade”, a resistência passiva ou as escolhas ecléticas das mulheres angolanas acabaram por condicionar as suas características, levando, por exemplo, à inclusão da medicina curativa nos dispensários.

Rompendo com abordagens estanques da realidade, o livro contempla por último a questão da emigração de angolanos para as colónias limítrofes, analisando as ansiedades e as respostas políticas que o despovoamento por essa via gerou entre os funcionários da administração colonial portuguesa. Não se conhecia a real dimensão da saída de angolanos para trabalhar em minas, plantações e infraestruturas de territórios vizinhos, em busca de melhores condições de vida, e para evitar impostos mais altos e o trabalho forçado na colónia portuguesa. Embora parcial e impreciso, o conhecimento demográfico sobre esses trânsitos reforçou os medos quanto ao declínio da população e do prestígio de Portugal como potência colonial e conduziu à adoção de medidas pela administração colonial em Angola que procuravam evitar esses fluxos, como a redução de impostos, o reforço do controlo administrativo e a instalação de missões católicas nas regiões fronteiriças.

Entre os méritos dessa obra, destaco a riqueza e diversidade da bibliografia, das fontes impressas e dos arquivos consultados, muitos deles inéditos, em seis países e quatro línguas; a aplicação consistente da perspetiva comparativa e transimperial – Coghe não se limita a olhar para Angola, vai fazendo comparações com o que se passava nas outras colónias portuguesas em África, no Portugal metropolitano e noutros impérios coloniais; a capacidade de combinar contributos de diferentes subdisciplinas da história; a solidez, pertinência e novidade da sua análise. A obra aproveita a todos os que se interessam pela história de África, da saúde, da demografia e das migrações, e é um exercício exímio de história transimperial da circulação do conhecimento (médico e demográfico), a partir de um local – a colónia portuguesa de Angola – ainda largamente inexplorado. Embora Coghe não dialogue com a recente historiografia sobre diplomacia científica ( Adamson, Lalli, 2021 ) para problematizar as conexões entre a produção de conhecimento sobre a doença do sono e a competição entre impérios em África nas primeiras décadas do século XX, o seu trabalho inclui um rico material empírico para o efeito.
